# Current status and advances in ultrasound evaluation of neovascularization within carotid artery plaques: a systematic review

**DOI:** 10.1186/s12947-025-00356-0

**Published:** 2025-09-01

**Authors:** Yang Yang, Fangqin Liu, Jiaojun Yan, Yunhao Luo, Qiuyun Huang, Lang Qiao

**Affiliations:** 1 School of Medical and Life Sciences, Chengdu University of Traditional Chinese Medicine, Chengdu, China; 2 Department of Ultrasound, Chengdu Sixth People’s Hos pital, Chengdu, China; 3 Department of Ultrasound, Sichuan Integrative Medicine Hospital, No.51, Section 4, Renmin South Road, Chengdu, 610041 China

**Keywords:** Intraplaque neovascularization (IPN), Superb micro-vascular imaging (SMI), Contrast-enhanced ultrasound (CEUS), Plane wave ultra-sensitive blood flow imaging (Angio PLUS), Ultrasound-targeted microbubble destruction (UTMD), Ultrasound super-resolution imaging (SRI)

## Abstract

Vulnerable plaques are significant risk factors for acute ischemic events, and intraplaque neovascularization (IPN) is an important indicator for evaluating plaque vulnerability. This review summarizes the importance of IPN in the assessment of carotid plaque vulnerability, the current status of ultrasound examination of IPN, and the technical advancements in ultrasound imaging of IPN, These techniques include: Superb micro-vascular imaging; Contrast-enhanced ultrasound; Plane wave ultra-sensitive blood flow imaging; Ultrasound-targeted microbubble destruction; Ultrasound Super-Resolution Imaging. Aiming to provide a reference for the prevention and treatment of ischemic cardiovascular and cerebrovascular events.

Vulnerable plaques are prone to rupture and thrombus formation, leading to acute ischemic events. When the arterial intima is damaged and an inflammatory response occurs, a large number of cytokines cause monocytes to adhere to endothelial cells. Through processes such as budding, migration, proliferation, and matrix remodeling, IPN is formed. IPN is characterized by an immature structure and an incomplete basement membrane, making it susceptible to rupture and bleeding [1]. Studies have shown that the density of IPN in vulnerable plaques is twice that of stable plaques, and the IPN density in ruptured plaques is four times that of stable plaques [2]. Therefore, IPN is a key indicator for assessing plaque stability.

Currently, the main diagnostic methods for IPN include ultrasound, magnetic resonance imaging, positron emission tomography-computed tomography, and optical coherence tomography, among which contrast-enhanced ultrasound and high-resolution vascular wall magnetic resonance imaging are considered the second gold standard, next to histopathology. Ultrasound is characterized by its convenience and cost-effectiveness, and it has significant advantages in the screening, diagnosis, and follow-up of arterial diseases. As a window for clinical research on atherosclerotic diseases, the assessment of carotid plaques is highly valued in the diagnosis and treatment of cardiovascular diseases. This review focuses on the ultrasound evaluation of IPN in carotid plaques.

## Current status of ultrasound detection of IPN

### SMI diagnosis of IPN

Superb micro-vascular imaging (SMI) is based on color Doppler technology and utilizes an adaptive signal data computation method to remove clutter and motion artifacts while preserving low-velocity blood flow signals [3], thereby enabling the visualization of IPN.

The pathological results following carotid endarterectomy show good consistency with SMI diagnosis of IPN, and the SMI grading is positively correlated with the histopathological microvascular density [4]. Multiple studies [5–7] have demonstrated that the detection rate of IPN and the SMI grading in vulnerable plaques are significantly higher than those in non-vulnerable plaques, with grade 2 and 3 blood flow being predominant. A meta-analysis [8] involving 605 patients with carotid stenosis confirmed that patients with IPN detected by carotid SMI are more likely to experience ischemic stroke or transient ischemic attacks, and those with ischemic events have higher SMI scores [9]. Additionally, the number of IPNs can change over time and with treatment [10].

**Fig. 1:**
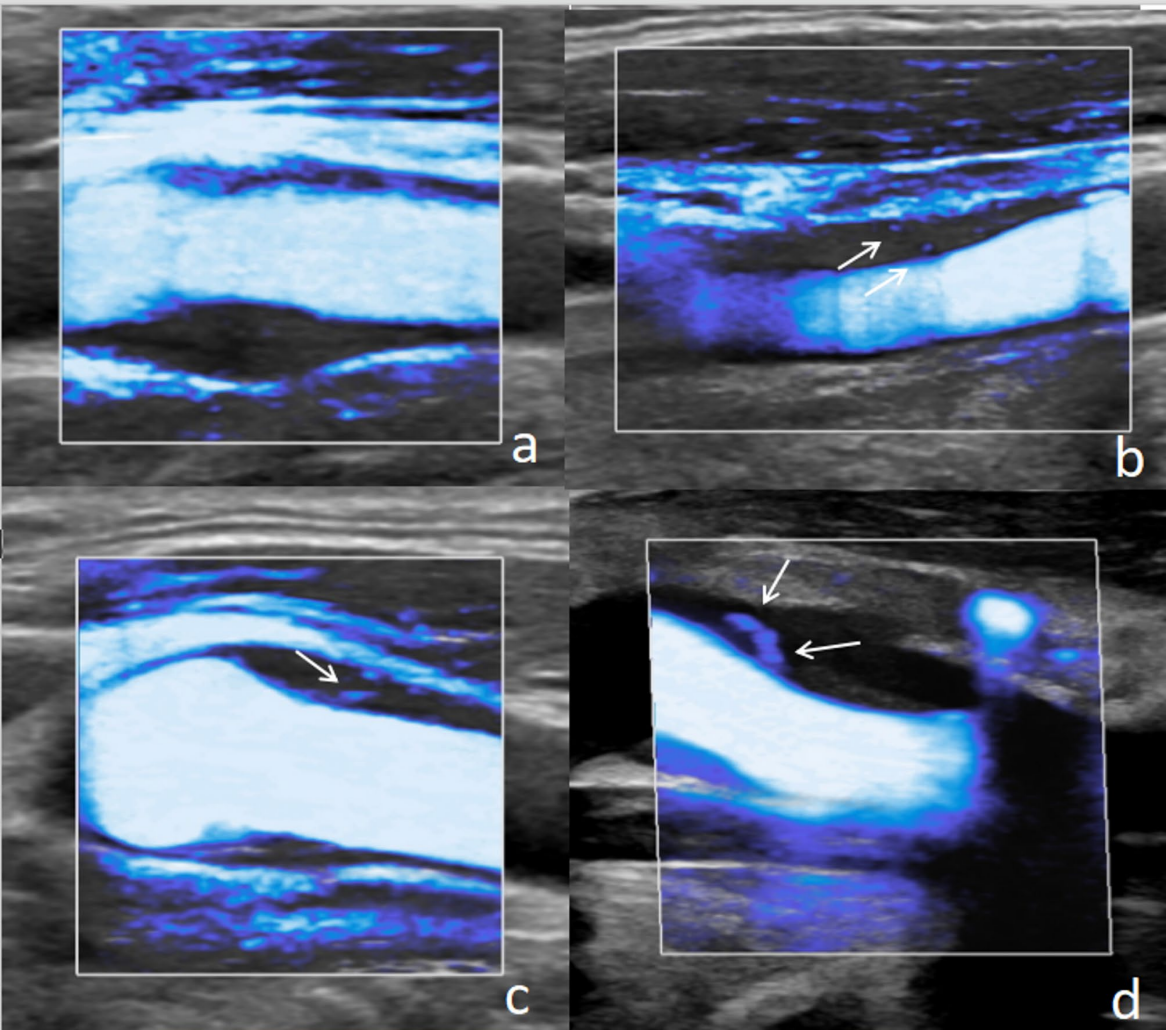
4 Category Classification of IPN by SMI. The 4-category Classification of IPN by SMI is illustrated as follows: **a**: Grade 0: No blood flow signal within the plaque. **b**: Grade 1: One or several punctate blood vessels visible within the plaque (indicated by arrows); **c**: Grade 2: Punctate blood vessels and 1–2 linear blood vessels detected (indicated by arrows); **d**: Grade 3: Multiple linear blood vessels visible within the plaque, with most penetrating through the plaque (indicated by arrows)

Currently, SMI detection of IPN is limited to semi-quantitative grading, with diverse grading methods (see Table 1) and no unified standard. However, due to its non-invasive and reproducible nature, and relatively simple operation, SMI can be routinely used for detecting IPN in vulnerable plaques and assessing clinical therapeutic effects. For semi-quantitative analysis, it is recommended to refer to the 4-category standard presented in Table 1 to align with contrast-enhanced ultrasound examination, As shown in Fig. 1. It should be noted that some manifestations of IPN in SMI are similar to microcalcifications within the plaque, and the results are highly subjective. However, IPN can change with the cardiac cycle.

**Table 1:** Semi-Quantitative classification of IPN by SMI

Classification Method	Specific Classification	Author	Year
2-category	Grade 0: Negative; Grade 1: Positive	Oura K [12], et al	2018
3-category	Grade 0: No blood flow detected; Grade 1: Less than 4 punctate blood flow signals at the plaque base or shoulder; Grade 2: Multiple short linear, linear, or tree-like neovascularizations within the plaque.	Li, M. [11], et al	2024
	Grade 0: No blood flow signal within the plaque; Grade 1: Blood flow signal detected at the shoulder or base of the plaque; Grade 2: Blood flow signals detected at both the shoulder and base of the plaque.	Zhang H [13], et al	2017
	Grade 0: No neovascularization within the plaque; Grade 1: One or two short, rod-like or punctate vessels present only at the base of the plaque; Grade 2: Three to four punctate vessels scattered within the plaque, or the presence of a longer vessel (length greater than the radius of the plaque).	Michal S [14], et al	2014
4-category	Grade 0: No blood flow signal within the plaque. Grade 1: One or several punctate blood vessels visible within the plaque. Grade 2: Punctate blood vessels and 1–2 linear blood vessels detected. Grade 3: Multiple linear blood vessels visible within the plaque, with most penetrating through the plaque	Chen X [6], et al	2020
5-category	Grade 0: No blood flow signal within the plaque or at the base. Grade 1: Blood flow signal detected at the base of the plaque. Grade 2: Blood flow signal detected at the shoulder of the plaque. Grade 3: Blood flow signal moving towards the core of the plaque. Grade 4: Extensive blood flow signal within the plaque	Zamani M [15], et al	2019

### Contrast-enhanced ultrasound (CEUS) for diagnosis of intraplaque neovascularization (IPN)

Contrast-enhanced ultrasound (CEUS) is primarily used to enhance the intensity of blood scattering signals through intravenous or subcutaneous injection of contrast agents, providing pure blood pool imaging. SonoVue (Bracco), a commonly used ultrasound contrast agent, has microbubbles with a diameter range of 1–10 μm that can act as red blood cell tracers. This allows for real-time visualization of the distribution and density of microvasculature within plaques [16]. CEUS can accurately display IPN within carotid artery plaques and is considered the second gold standard for assessing plaque vulnerability. However, CEUS is currently limited in its clinical application due to its high cost and the lack of standardized operating procedures and diagnostic consistency.

#### CEUS findings of IPN

Contrast agents can simulate the hemodynamics of red blood cells, thereby visualizing the size, quantity, and location of blood vessels [17]. Multiple studies [18–20] have demonstrated that carotid plaques with enhanced manifestations on angiography are associated with the presence of IPN, and the intensity and area of contrast enhancement are consistent with the number, density, and area of IPN. Dong et al. [21] conducted a meta-analysis to investigate the diagnostic performance of CEUS compared with histopathological examination for the evaluation of carotid IPN. They found that CEUS exhibited high sensitivity and specificity for the assessment of carotid IPN, with values of 83% and 77%, respectively.

Contrast agent microbubbles typically exhibit sparse punctate enhancement from the periphery to the interior of the plaque. The enhancement patterns vary among plaques with different echogenicities and compositions. Hypoechoic plaques show the highest enhancement intensity, followed by mixed-echo plaques [18, 22], while hyperechoic plaques exhibit the lowest enhancement [23]. Homogeneous plaques generally have higher enhancement than heterogeneous plaques. Moreover, contrast agent microbubbles can directly adhere to the damaged endothelium in inflamed regions and can be phagocytosed by neutrophils and monocytes, remaining intact in the body for over 30 min [24]. Due to the significant influence of shear stress from blood flow on the proximal end of the plaque, this region is more susceptible to IPN formation. Therefore, enhancement on carotid plaques is more pronounced at the proximal end. The enhancement intensity in the shoulder of the plaque was correlated with the density of new vessels, and the enhancement intensity in the shoulder of the plaque with rupture was higher than that of the plaque without rupture, and the enhancement intensity in the symptomatic plaque was higher than that of the asymptomatic plaque [25].

#### Semi-quantitative analysis of CEUS

**Fig. 2:**
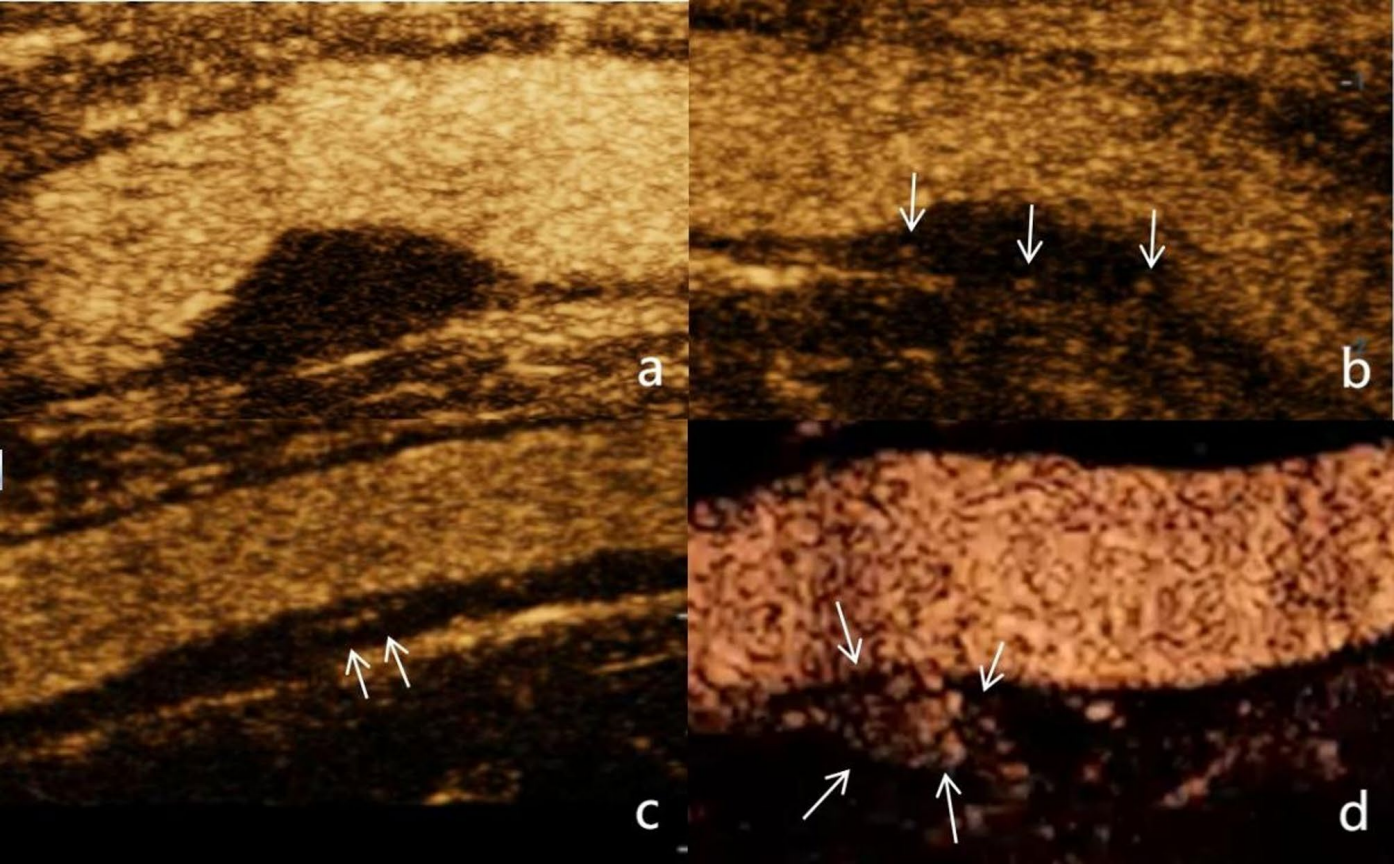
Four-Category Classification of IPN Enhancement on CEUS

Currently, there is a variety of semi-quantitative grading systems for IPN using CEUS, as shown in Table 2. The first of the four classifications is recommended as shown in Fig. 2. The higher the IPN grade, the more unstable the plaque tends to be ([26]– [27]). In clinical observational studies, high-grade IPN is closely associated with the occurrence of ischemic stroke and cardiovascular events [28]. The higher the IPN grade of carotid plaque, the higher the risk of cardiovascular events ([29]– [30]). Semi-quantitative classification of IPN by CEUS is beneficial for the prevention and treatment of ischemic events, as well as for prognostic assessment.

The four-category classification of IPN enhancement on CEUS is illustrated as follows: a:Grade 0: No enhancement within the plaque; b:Grade 1: Focal punctate enhancement within the plaque (indicated by arrows); c:Grade2: Intermediate between Grade 1 and Grade 3, with punctate and 1–2 linear enhancements (indicated by arrows); d:Grade 3: Linear enhancement within the plaque, which may traverse or mostly traverse the plaque, or signs of blood flow are present (indicated by arrows).

**Table 2:** Semi-Quantitative classification of IPN by CEUS

Classification Method	Specific Classification	Author	Year
3-category	Grade 0: No visible microbubbles within the plaque; Grade 1: Limited to moderate number of microbubbles visible within the plaque; Grade 2: Diffuse microbubbles visible within the plaque	Schinkel AFL [30], et al.	2020
4-category	Grade 0: No enhancement within the plaque; Grade 1: Focal punctate enhancement within the plaque; Grade 2: Intermediate between Grade 1 and Grade 3, with punctate and 1–2 linear enhancements; Grade 3: Linear enhancement within the plaque, which may traverse or mostly traverse the plaque, or signs of blood flow are present	Shah F( 31], et al.	2007
	Grade 0: No enhancement within the plaque; Grade 1: Enhancement limited to the adventitia or periadventitial region; Grade 2: Enhancement at the plaque shoulder or adventitial side; Grade 3: Extensive enhancement within the plaque	Johri M A [32], et al.	2017
	The longitudinal section of the carotid plaque was divided into four regions: base, proximal shoulder, distal shoulder, and apex. The semi-quantitative grading of contrast agent perfusion on CEUS was defined as follows: Grade 1, contrast agent perfusion in one region; Grade 2, contrast agent perfusion in two regions; Grade 3, contrast agent perfusion in three regions; Grade 4, contrast agent perfusion in all four regions	Li L [33], et al.	2018
5-category	Grade 0: No obvious enhancement within the plaque; Grade 1: Enhancement in either the shoulder or the base; Grade 2: Enhancement in both the shoulder and the base; Grade 3: Enhancement extending towards the center of the plaque; Grade 4: Diffuse enhancement throughout the plaque	Zamani [34], et al.	2020
	Grade 0: No enhancement; Grade 1: No enhancement within the plaque, with enhancement in the adventitia; Grade 2: Focal enhancement within the plaque, presenting as scattered punctate or minimal enhancement; Grade 3: Linear enhancement extending into the plaque; Grade 4: Diffuse enhancement throughout the plaque	SONG ZE ZHOU [35], et al.	2015

#### Quantitative analysis of CEUS

Quantitative analysis in contrast-enhanced ultrasound (CEUS) involves the use of specialized software to convert perfusion information of IPN into specific numerical data. Apart from the quantitative analysis software equipped with the ultrasound instrument, some commercial software can also quantitatively evaluate IPN within the plaque. For instance, Vuebox (Bracco, Italy) and QLAB (Philips, the Netherlands) are such software. These quantitative software can obtain parameters such as peak time (TP), mean transit time (MTT), area under time-strength curve (AUCt), peak intensity ratio (Peak), peak intensity ratio value (PI), and enhanced intensity (EI) of the region of interest. Multiple studies have demonstrated that IPN density is negatively correlated with TP and MTT, and positively correlated with AUCt, PI, and EI ([36]– [37]). In other words, for vulnerable plaques, lower values of TP and MTT, and higher values of AUCt, Peak, PI, and EI are indicative of increased plaque instability. Furthermore, larger values of PI and EI are associated with larger cerebral infarct sizes and worse prognosis [38]. Patients with recurrent acute cerebral infarction have higher EI and Peak values compared to those without recurrence [39]. Quantitative analysis of CEUS can serve as a robust indicator for assessing the risk of ischemic events.

### Combined diagnosis of IPN by CEUS and SMI

Multiple studies [40–42] have demonstrated a positive correlation between the results of IPN detection using SMI and CEUS in carotid plaques. Although CEUS has slightly higher diagnostic efficacy than SMI, the two modalities show good consistency and similar accuracy. For instance, a study [26] reported that the sensitivity, specificity, and accuracy of SMI and CEUS for detecting vulnerable carotid plaques were 82.4%, 80%, and 81.8% for SMI, and 94.1%, 60%, and 86.3% for CEUS, respectively. When combined, SMI and CEUS achieved higher diagnostic performance, with sensitivity, specificity, and accuracy reaching 94.1%, 80%, and 90.9%, respectively. The diagnostic efficacy of SMI combined with CEUS in evaluating vulnerable plaques is superior to that of SMI or CEUS detection alone. The combined application of CEUS and SMI can enhance the sensitivity, specificity and accuracy of diagnosing the vulnerability of carotid plaques. Moreover, both CEUS and SMI techniques have their limitations at present. CEUS may take 10–20 min from the beginning of preparation of contrast media and equipment to the completion of imaging, while SMI does not need to prepare contrast media and injection equipment, and the preparation time is relatively short, usually only 1–2 min, but the exact time ultimately depends on the proficiency of the operator and the specific requirements of the examination. SMI only has semi-quantitative analysis and is subject to subjective influence. The cost of ultrasound contrast agent is high and there is a lack of standard operating procedures and diagnostic consistency. The combined application of CEUS and SMI can provide more reliable IPN evaluation. However, a unified semi-quantitative grading standard for IPN needs to be established, which has significant application value.

**Table 3:** Advantages and limitations of SMI versus CEUS

Feature	SMI	CEUS
Principle	Based on color Doppler technology, an adaptive signal data calculation method was used to remove clutter and motion artifacts, while retaining low-velocity blood flow signals.	Pure blood pool imaging was provided by using intravenous microbubble contrast agent to enhance the scattered blood signal.
Advantages	Non-invasive examination, without injection of contrast material, is safer; More quickly; There were few motion artifacts.	Can be quantified.
Limitations	At present, it is limited to semi-quantitative grading and lacks unified quantitative standards; Neovascularization with very low flow rates (< 0.4 cm per second) may not be clearly visualized.	There are many contraindications, such as allergy, acute heart failure, endocarditis, right-to-left shunt, and unstable angina; The examination takes a long time.
Scope of application	It is suitable for routine screening and initial evaluation of vulnerable plaques, especially when non-invasive tests are required.	It is suitable for detailed assessment and study of plaque vulnerability, especially when quantitative analysis is required.

## Recent advances in ultrasound technology for IPN detection

### Angio PLUS for the diagnosis of IPN

Angio Planwave Ultrasensitive Imaging (Angio PLUS), abbreviated as AP technology, is capable of detecting low-velocity blood flow signals below 100 μm without the need for contrast agents, earning it the moniker “contrast-free angiography.” By introducing an additional spatial frequency axis based on two-dimensional filtering, it enables the analysis of echo signals in a three-dimensional spatial mode. This allows for the detection of blood flow without compromising sensitivity, temporal resolution, or spatial resolution, and also enables the differentiation of blood flow direction [43].

The current Angio PLUS scoring system for IPN is as follows: 0 points for no IPN blood flow signals detected; 1 point for a few punctate IPN blood flow signals (< 4) detected at the plaque shoulder or base; 2 points for multiple short linear, linear, or arborizing IPN blood flow signals detected. The AP score is significantly correlated with the number of IPN detected histologically [44]. Angio PLUS shows high consistency with SMI and CEUS in the detection rate and grading of IPN. However, AP technology has an advantage over SMI in detecting IPN at the plaque shoulder [45], while IPN with a CEUS score of 1 may be less clearly visualized by AP technology. AP technology can conveniently, non-invasively, early, and rapidly screen for IPN, which is helpful for identifying vulnerable plaques and guiding the prevention and treatment of ischemic events in clinical practice. Currently, research on AP technology is limited, but it holds broad prospects for future investigation and application.

### UTMD for the diagnosis and treatment of IPN

Ultrasound-Targeted Microbubble Destruction (UTMD) is a technique that involves encapsulating bioactive drugs or target genes within ultrasound microbubble contrast agents, which are then delivered to the target organs or tissues via the circulatory system. Upon exposure to ultrasound, the microbubbles are destroyed, generating cavitation and sonoporation effects. These effects play crucial roles in thrombolysis, drug delivery, and gene therapy [46]. Ligands such as P-selectin, vascular adhesion molecules, lectin-like oxidized low-density lipoprotein receptors, and von Willebrand factor can be conjugated to microbubbles for targeted imaging of atherosclerotic plaques [47]. Targeted microbubbles have been shown to provide superior imaging capabilities for carotid atherosclerotic plaques compared to traditional bare microbubbles [48]. The mechanical damage induced by the cavitation effect of ultrasound can act on the vascular wall, activating the coagulation mechanism and inducing damage and disruption of IPN, thereby reducing IPN and improving plaque stability. The addition of ultrasound contrast agents can enhance the cavitation effect, thereby further strengthening the therapeutic efficacy [49]. For instance, a study [50] demonstrated that after 24 h of treatment with ultrasound combined with microbubbles in the late atherosclerotic plaque model of mice, the density of IPN within the plaque was significantly reduced by 84%, and the number of immature IPN within the plaque was also significantly decreased by 90%, while there was no obvious change in normal tissues. Therefore, UTMD has more advantages over traditional contrast agents in the display of IPN, and can effectively reduce or control IPN within the plaque, improve plaque stability. At present, UTMD research is in its early stage and is only limited to preclinical research. However, compared with surgery, UTMD can be used as a new entry point in the treatment of plaque and reduce the occurrence of ischemic events with less damage, which has great development prospects.

### SRI for the diagnosis of IPN

Ultrasound Super resolution Imaging (SRI) technology, also known as ultrasound localization microscopy (ULM), refers to a high-frequency acoustic imaging technique that integrates ultrasound localization microscopy, super-resolution wavefront imaging, and structural imaging technologies. SRI is based on ultrasound contrast imaging and utilizes tracking of individual microbubbles for non-invasive identification of microvessels with diameters less than 10 μm. It not only enables the visualization of microvascular morphology but also calculates parameters such as microvascular flow rate (MFR) and microvessel density (MVD) ([51]– [52]). Preclinical studies of SRI started with the microflow images and related parameters of mouse ear [53], rabbit liver [54], and rabbit lymph node [55]. In 2018,Opacic et al. [56] performed SRI imaging of breast cancer lesions on human body for the first time. At present, SRI has been applied to various organs and tissues of human body, and the microblood flow calibration of thyroid [57], breast [52], eye [58], liver [59], kidney [60] and other important tissues and organs has been realized. Additionally, it can quantify tumor microvascular characteristics and vascular tortuosity indices, thereby improving the diagnostic accuracy of differentiating benign from malignant tumors and assessing therapeutic efficacy [52]. SRI related studies for carotid IPN are few. The latest studies show that SRI can obtain the blood flow velocity within the plaque and the microvascular flow is greater in the systolic phase than in the diastolic phase [61]. However, there are few quantitative data in this article, and more detailed research data are needed. SRI represents a highly precise ultrasound imaging modality with significant potential for studying the structural and hemodynamic features of IPN.

## Conclusion

SMI is non-invasive and easy to perform but is susceptible to subjective interpretation. It can serve as a routine screening method for the diagnosis of IPN. CEUS acts as a secondary gold standard for assessing plaque vulnerability, holding an important position with its unparalleled capability for quantitative analysis. However, the lack of standardized operating procedures and diagnostic consistency, coupled with its relatively high cost, limits its application in large-scale screening of IPN. Angio PLUS is non-invasive and shows good correlation with SMI and CEUS. However, there is still room for improvement in its widespread application and quantitative analysis. UTMD provides better visualization of IPN compared with traditional CEUS and holds greater therapeutic potential. Nevertheless, its clinical application remains limited, and it has significant value for further research and application. SRI offers higher resolution for microvessel visualization and enables quantitative analysis of IPN, showing great potential for IPN detection.

Ultrasound examination has the advantages of being real-time, cost-effective, easy to perform, and highly reproducible. It can qualitatively and quantitatively assess plaque vulnerability and holds unique advantages in evaluating plaque vulnerability and therapeutic efficacy. Ultrasound is the preferred imaging modality for screening and diagnosing vulnerable plaques, facilitating the early warning, diagnosis, treatment, and follow-up of ischemic events. However, each technique has its strengths and limitations, and all have significant potential for development and research.

## Data Availability

No datasets were generated or analysed during the current study.

## Abbreviations

IPN: Intraplaque neovascularization
SMI: Superb micro-vascular imaging
CEUS: Contrast-enhanced ultrasound
Angio PLUS: Plane wave ultra-sensitive blood flow imaging
UTMD: Ultrasound-targeted microbubble destruction
SRI: Ultrasound super-resolution imaging
TP: Peak time
MTT: Mean transit time
AUCt: Area under time-strength curve
Peak: Peak intensity ratio
PI: Peak intensity ratio value
EI: Enhanced intensity
